# Chuna (or Tuina) Manual Therapy for Musculoskeletal Disorders: A Systematic Review and Meta-Analysis of Randomized Controlled Trials

**DOI:** 10.1155/2017/8218139

**Published:** 2017-12-26

**Authors:** Nam-Woo Lee, Gee-Heon Kim, In Heo, Koh-Woon Kim, In-Hyuk Ha, Jun-Hwan Lee, Eui-Hyoung Hwang, Byung-Cheul Shin

**Affiliations:** ^1^School of Korean Medicine, Pusan National University, Yangsan 50612, Republic of Korea; ^2^Korean Medicine Clinical Research Center, Korean Medicine Hospital, Pusan National University, Yangsan 50612, Republic of Korea; ^3^Department of Korean Rehabilitation Medicine, Kyung Hee University, Seoul 02447, Republic of Korea; ^4^Jaseng Spine and Joint Research Institute, Jaseng Medical Foundation, Seoul 06017, Republic of Korea; ^5^Clinical Research Division, Korea Institute of Oriental Medicine, Daejeon 34054, Republic of Korea; ^6^Korean Medicine Life Science, University of Science & Technology (UST), Campus of Korea Institute of Oriental Medicine, Daejeon 34054, Republic of Korea; ^7^Division of Clinical Medicine, School of Korean Medicine, Pusan National University, Yangsan 50612, Republic of Korea; ^8^Spine & Joint Center, Korean Medicine Hospital, Pusan National University, Yangsan 50612, Republic of Korea

## Abstract

**Objective:**

To review the literature and systematically evaluate the effectiveness of Chuna (or Tuina) manual therapy (C[T]MT) on pain and function for musculoskeletal disorders.

**Methods:**

We searched 15 English, Chinese, Japanese, and Korean databases using relevant keywords. All randomized controlled trials (RCTs) of C(T)MT for musculoskeletal disorders were considered, and we limited analyses to studies with a low-risk bias for randomization and/or allocation concealment.

**Results:**

Sixty-six RCTs with 6,170 participants were included. One sham-controlled RCT showed that C(T)MT relieved pain more effectively than a sham control (SMD −3.09 [−3.59, −2.59]). For active-controlled RCTs, pooled meta-analysis showed that C(T)MT had statistically significant effects on pain reduction, especially compared to traction (*P* < 0.00001), drugs (*P* = 0.04), and physical therapies (*P* < 0.0001). For functional improvement, combined effects of C(T)MT with drugs (*P* = 0.04) and traction (*P* = 0.05) also showed similar positive effects.

**Conclusions:**

This systematic review suggests that C(T)MT is safe and effective for pain reduction and functional improvement for musculoskeletal diseases; however, the evidence for functional improvement was not as strong as for pain reduction. For future studies, high-quality RCTs such as sham-controlled studies with standardized interventions are needed to provide sufficient evidence on the effects of C(T)MT for musculoskeletal diseases. Protocol registration number is CRD42016038307 04/07/2016.

## 1. Introduction

Musculoskeletal disorders present an increasing global health care problem, being the number one self-reported medical condition in the United States (US) according to the National Health Interview Survey (NHIS) in 2012. These disorders are the most common cause of chronic severe pain and physical dysfunction and they affect hundreds of millions of people around the world. The economic impact of these conditions in the US is also astounding, costing the US an estimated $874 billion in treatment costs and lost wages annually, or 5.7% of the 2011 Gross Domestic Product [1]. The neck and back are the most common areas of musculoskeletal disorders, followed by the upper limbs and lower limbs [2]. Beyond these statistics, when we look at the quality of life, the situation is unlikely to get better due to current aging trends and the high activity levels of elderly population [1]. Manipulation approaches are becoming increasingly popular for the treatment of musculoskeletal disorders. Almost 30% of people with neck pain or dysfunction have used manipulation methods to treat their problems [3].

Chuna (Korea) or Tuina is a manipulation treatment that addresses biomechanical function, diagnostics, pathology, and theories to balance orthopaedic structure and function. Chuna or Tuina works along the meridians throughout the body, corrects the displacement of the structures, and prescribes exercises based on symptoms and the results of a functional assessment. It represents techniques such as thrust, mobilization, distraction of the spine and joints, visceral manipulation, soft tissue release, craniosacral therapy, and the diaplasis technique [4]. Traditional Chuna (Korea) is based on Traditional Chinese Tuina but represents the combination of traditional practice and modern scientific knowledge in fields such as anatomy, pathology, and physiology. Traditional Korean Chuna has become Modern Korean Chuna by integrating Chinese Tuina, American chiropractic practice and osteopathy, and Japanese manipulation techniques. A substantial number of randomized controlled trials (RCTs) have shown that Chuna or Tuina is effective for several diseases, such as musculoskeletal [5], neuropsychiatric [6], and cardiovascular disorders [7]. Among these diseases, musculoskeletal disorders are the most common diseases.

So far, we have found 27 systematic reviews about these diseases. Of these, 20 studies were about musculoskeletal disease [8–27] such as neck pain, back pain, and shoulder pain. Two were about neuropsychiatric diseases [28, 29] and 5 were about other diseases [30–34], such as hypertension and cancer pain. However, many of these reviews do not adhere to Preferred Reporting Items for Systematic reviews and Meta-Analyses (PRISMA) reporting guidelines [35], and many were not conducted systematically. Therefore, this study aimed to summarize the current evidence on Chuna (or Tuina) manual therapy for relief of pain and improvement of function for musculoskeletal disorders, with adherence to the PRISMA reporting guidelines.

## 2. Methods

### 2.1. Data Sources and Searches

The following electronic databases were searched up to December 2016. We searched 4 worldwide databases (PubMed, Ovid LWW Medline, EMBASE, and Cochrane Library), 3 Chinese databases (China National Knowledge Infrastructure [CNKI], Wanfang, and VIP), 1 Japanese database (J-stage), and 7 Korean databases (Korean Medical Database [KMBASE], Korean Studies Information Service System [KISS], National Discovery for Science Leaders [NDSL], Database Periodical Information Academic [DBpia], Korean National Assembly Digital Library [KNADL], Oriental Medicine Advanced Searching Integrated System [OASIS], and Korean Traditional Knowledge Portal [KTKP]).

The search terms used for PubMed were as follows: (((Tuina) OR Chuna)) AND ((((Randomized Controlled Trial) OR Randomised Controlled Trial) OR rct) OR Randomized) OR Randomised. For other databases, the search terms were slightly modified but still included terms such as (Tuina OR Chuna) AND (Randomised Controlled Trials). Furthermore, the references regarding our articles were manually searched for further relevant articles.

### 2.2. Study Selection

#### 2.2.1. Inclusion Criteria

This systematic review included parallel or crossover RCTs that evaluated the effects of Chuna (or Tuina) manual therapy (C[T]MT) on pain and function for musculoskeletal diseases.

Patients who reported any kind of musculoskeletal disorders were eligible for inclusion. This review included patients regardless of gender, age, and race. The patients with musculoskeletal disorders were classified according to affected area (spine, upper extremity, and lower extremity) and then subclassified according to exact diagnosis.

For interventions, we included C(T)MT intervention only and excluded other types of manual therapy. Studies that assessed the combined effects of Chuna (or Tuina) plus other interventions were also considered when the identical intervention was administered to both the Chuna (or Tuina) group and the control group.

For control groups, we considered sham treatment or other active interventions, except other kinds of Chuna (or Tuina). The sham Chuna (or Tuina) treatment(s) were regarded as those that employed the same/similar Chuna techniques, but with no active components. Other interventions included traction, physical therapy, drug therapy, and surgery.

We only included pain and function outcome measurements for musculoskeletal conditions. For pain, we used a visual analogue scale (VAS) and a numerical rating scale (NRS). For function, we used the neck pain disability index (NDI), the Oswestry disability index (ODI), and the Constant-Murley score (CMS). Additionally, we included complications to assess safety outcomes.

Eventually, we included three types of study model: (1) Chuna (or Tuina) versus sham, (2) Chun (or Tuina) versus other interventions, and (3) Chuna (or Tuina) plus other interventions versus same other interventions.

#### 2.2.2. Exclusion Criteria

Regarding types of research, we excluded quasi-RCTs that did not allocate participants to a treatment group in a truly random way, for example, according to hospital record number or alternation and date of birth, or RCTs that did not clearly report that a random method was used and those that adopted inappropriate methods.

For Chuna (or Tuina) manual interventions, we excluded studies that employed other kinds of manual treatments, or those in which there was no clear description of methods.

Trials comparing different types of Chuna (or Tuina) were excluded, because the effectiveness of Chuna (or Tuina) compared to other interventions could not be assessed.

We did not include patients with musculoskeletal disorders found to be caused by psychogenic and neurologic conditions, or other reasons, except for musculoskeletal aetiologies.

### 2.3. Data Extraction

Two independent reviewers (Nam-Woo Lee and Gee-Heon Kim) screened the titles and abstracts for potentially eligible studies identified by the primary search and then reviewed the full texts to evaluate their final eligibility. All Chinese articles were reviewed by Nam-Woo Lee who graduated from Beijing University of Chinese Medicine. All English and Korean articles were reviewed by Gee-Heon Kim. The two authors cross-checked each other's articles and if there were any disagreements regarding extracted data, we contacted the original authors via e-mail or telephone to request additional information.

After selecting articles for inclusion, we extracted the following data: authors, publication year, types of disease, study design, sample size, treatment and follow-up duration, interventions, outcome measures on pain and function, and the main results (Table 3). We also extracted the following data regarding musculoskeletal conditions and study design (Table 1).

### 2.4. Assessment of Risk of Bias (ROB)

Quality assessment was conducted using the Cochrane risk of bias criteria tools [36]. We ranked each item into three levels: “low (green),” “unclear (yellow),” or “high (red)” ROB. To gauge the participant blinding in sham control studies, we categorized the study as having a low ROB when blinding of patients was clearly expressed. To assess the ROB on outcomes, we concluded that a study had a low ROB if authors plainly reported that they blinded the outcome assessors or the outcome measure was assessed by blinded participants only. Studies were rated as having an unclear ROB if the outcome measures were built from both subjective and objective assessments, and we could not clearly judge whether the outcome assessor was blinded or not. Regarding the reporting of incomplete outcome data, a study was rated as having a low ROB if it satisfied three things: (1) the number of attrition cases and the causes were clearly reported in each group, (2) the attrition rates were similar between groups, and (3) the percentage of withdrawals and drop-outs did not exceed 20% in the short-term and 30% in the long-term follow-up period [36]. If there were no drop-outs in studies, they were rated as having a low ROB. When we confronted problems referring to the trial, we solved this problem by having a consensus-based discussion among reviewers.

### 2.5. Data Analyses

All outcome measurements were extracted as mean and standard deviation (or transformed) or total and events. The outcome measures at the end of the treatments were used in data pooling.

The risk estimates (relative risk: RR) were calculated for dichotomous data. For continuous data, standardized mean differences (SMDs) were employed because different scales were used for studies (e.g., VAS 0–10 or VAS 0–100). Weighted mean differences (WMDs) were used for continuous data if authors evidently reported that identical scales were used for the outcomes. Additionally, 95% confidence intervals (CIs) were calculated in the meta-analysis. For studies with more than one control group, we restricted our analyses to compare C(T)MT and control groups. The statistical heterogeneity was assessed using the *I*^2^ test. We determined that heterogeneity existed if *I*^2^ was above 50% [37]. To obtain more precise heterogeneity, we used a subgroup analysis by categorizing studies based on type of diseases, body parts, and various interventions. If heterogeneity continued, individual analysis was utilized. Additionally, our review used the random effect model to deal with heterogeneity that employs variation factors as correction weight. We analysed the RCTs with low ROBs for randomization and/or allocation concealment only and examined whether the estimate of the intervention effect was affected [38, 39]. Meta-analysis was performed using the Review Manager software (version 5.3 for Mac; the Nordic Cochrane Centre, Copenhagen, Denmark).

## 3. Results

### 3.1. Study Selection

**Figure 1:**
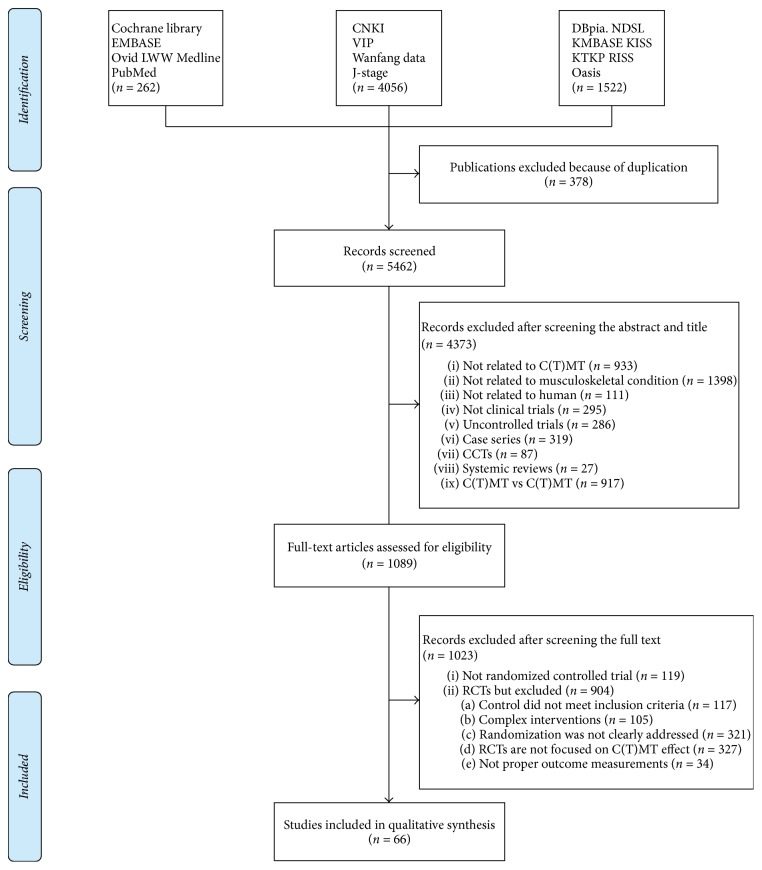
Flowchart of the RCT selection process. CCTs: controlled clinical trials; RCTs: randomized controlled trials; C(T)MT: Chuna (or Tuina) manual therapy.

Our search terms yielded 5,840 records. There were 262 from the Cochrane library, EMBASE, Ovid LWW Medline, and PubMed. There were 4,056 from CNKI, Wanfang data, VIP, and J-stage. There were 1,522 studies from domestic Korean databases and relevant journals. After removing duplicated studies, 5,462 records were screened. Based on the title and abstract, 4,373 records were excluded (Figure 1). Of these, 27 were systematic reviews related to C(T)MT and were analysed separately to find relevant studies. We retrieved and reviewed 1,089 full articles. After full text review, 1,023 records were excluded, 119 articles were not randomized clinical trials, and 904 did not meet the inclusion criteria due to several reasons that are summarized in Figure 1. Finally, a total of 66 RCTs (Chinese: *n* = 65; English: *n* = 1) were included in our review. Figure 1 shows a flow diagram of the literature search as recommended by PRISMA [35]. Details of the included studies are summarized in Table 3.

### 3.2. Study Characteristics

All RCTs (*n* = 66) and the data of 6,170 participants were included in the review. The number of participants in each group ranged from 11 to 200 in the C(T)MT group and from 11 to 200 in the control group. The study duration ranged from 1 day to 24 weeks. The number of sessions was 11.3 ± 8.1 sessions (range 1–36) and the length of each session was 25.3 ± 5.7 minutes (range 15–30). The follow-up time ranged from 1 day to 60 weeks.

Of the 66 RCTs, 1 RCT was C(T)MT versus sham C(T)MT [40], 48 RCTs were C(T)MT versus other active interventions [5, 41–87], and 17 RCTs were C(T)MT plus other active interventions versus same other interventions [88–104] (Tables 1 and 3).

The control therapies contained sham C(T)MT, block therapy, Chinese patent drugs, general rehabilitation treatment, intravenous injection, oral drugs, pharmacopuncture and surgical interventions in cases of fracture, physical therapy (including intermediate frequency therapy, micro current therapy, ultrasonic treatment, and TENS), traditional Chinese medicine, and traction (Table 3).

The types of diseases/disorders were very diverse and heterogeneous. Thus, we classified them according to body parts such as spine, upper extremity, and lower extremity (Table 1). The most common disorders were spine disorders (*n* = 42). Among them, 24 studies were for cervical spine [41–62, 88, 89], 14 studies were for thoracolumbar spine [63–70, 90–95], and 4 were classified as others such as scoliosis, sacrococcygeal pain, and ankylosing spondylitis [71, 72, 96, 97]. Studies about extremity diseases/disorders were classified into upper (*n* = 13) and lower extremity (*n* = 11), including 5 studies about shoulder lesions [5, 73, 74, 98, 99], 8 about arm and hand disorders [75–81, 100], 8 about knee problems [82–85, 101–104], and 2 about leg and foot disorders [86, 87]. One sham control study was a RCT that looked at overall musculoskeletal disorders, so it was not possible to classify it into a specific category [40]. Therefore, we have indicated the percentages for each part based on how the authors reported them in their study.

Outcome measures reported in the included studies were very diverse because of the various types of disease reported on. For pain, the McGill Pain Questionnaire (MPQ), the McGill Pain Questionnaire-Short Form (MPQ-SF), or a NRS, VAS, or visual numeric rating scale (VNRS) was used. For functional measurements, the clinical assessment scale for cervical spondylosis (CASCS), a NDI, an ODI, or a range of motion (ROM) or straight leg raising test (SLRT) was used. For both pain and function assessment, CMS, hospital for special surgery (HSS), or total score of symptoms and signs (TSS) was used, and activities of daily living (ADL) or SF-36 were used for quality of life (QOL) (Table 3).

### 3.3. Assessment of ROB

Most of the selected trials were judged as having a high ROB. The particulars of the ROB assessments are described in Figure 3. All 66 studies employed appropriate methods of sequence generation. For example, they employed a random number table, a coin toss, a randomisation code, or a computer random number generator. Group assignment was adequately concealed in 18 trials (27.3%), using sealed opaque envelopes or central allocation.

Of the 66 studies, only 3 RCTs [40, 44, 52] reported a proper description of participant blinding and assessor blinding. Participant blinding was performed in only one trial [40]. Double-blinding of the participants and practitioners did not occur. The outcome assessors were blinded in two trials [44, 52]. Both trials had independent assessors to evaluate outcome measurements.

Regarding incomplete outcome data, we evaluated 62 studies as having a low ROB. Many of them had no missing data or few missing data. In studies that had missing outcome data, the frequencies and causes for drop-outs in each group did not differ much. Moreover, the drop-out percentage in the short-term did not surpass 20%, and, in the long-term, the rate did not go over 30%. We could not calculate the drop-out rates of 4 trials [56, 91, 93, 102] because the numbers of participants were not reported in the results section.

For the selective outcome reporting, it was not possible to locate and study the protocols of any of the selected studies. In response, we discerned the ROB using the reported methods in each study. One study [44] had an unclear ROB because the authors failed to report each score of the test despite their claim to do so in the methods part. Only the total score was reported, without scores for each item. One study [56] had a high ROB because the authors did not include the incidence rate of complications in the results section, despite their promise to do so in the methods section.

### 3.4. Quantitative Data Synthesis

**Figure 2:**
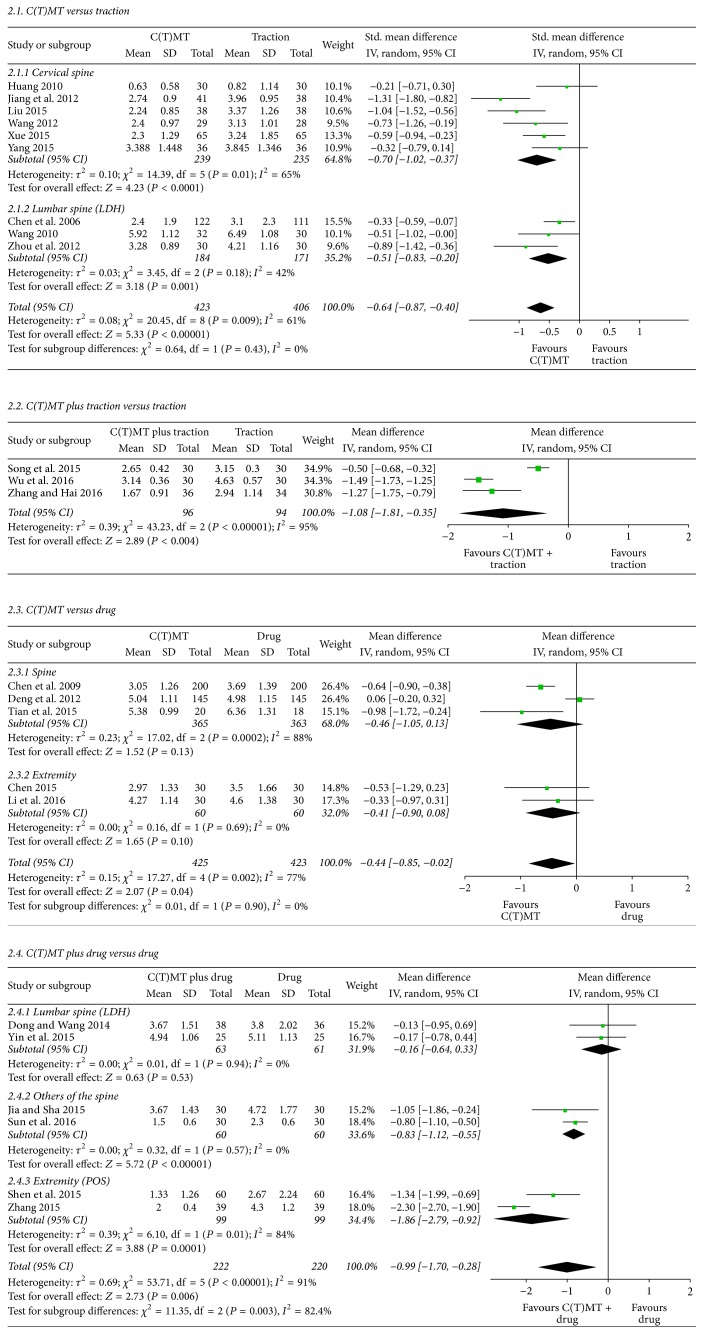
C(T)MT on pain outcomes (visual analogue scale) for musculoskeletal conditions. C(T)MT: Chuna (or Tuina) manual therapy; LDH: lumbar disk herniation; POS: periarthritis of shoulder.

**Figure 3:**
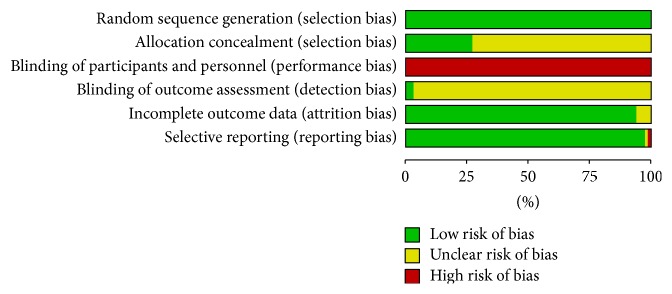
Risk of bias assessment.

The key outcomes from the included studies are provided in Figure 2 and Table 2.

#### 3.4.1. Effects of C(T)MT on Pain

*(1) Effects of C(T)MT versus Sham C(T)MT on Pain*. One RCT [40] assessed the effect of C(T)MT on pain versus sham C(T)MT for musculoskeletal conditions. The study showed a significant effect of C(T)MT on pain relief compared to sham C(T)MT. The meta-analysis also showed favourable effects of C(T)MT (*n* = 69; SMD, −3.09; 95% CI, −3.59 to −2.59; *P* < 0.00001; heterogeneity: NA; Table 2). The study by Sousa et al. [105] was excluded because the participants of the intervention group treated themselves with self C(T)MT, and treatment was not performed by a practitioner.

*(2) Effects of C(T)MT versus Traction on Pain*. Eight RCTs tested the effectiveness of C(T)MT compared to traction on pain relief. Among the 9 studies, 6 were for cervical diseases/disorders such as cervical spondylotic radiculopathy [44, 46, 49–51] and degenerative instability [60]. Three RCTs investigated lumbar disc herniation [63–65]. The meta-analysis showed favourable effects of C(T)MT on pain for cervical spondylotic radiculopathy (*n* = 474; SMD: −0.70; 95% CI −1.02 to −0.37; *P* < 0.0001; heterogeneity: *χ*^2^ = 14.39, *P* = 0.01, *I*^2^ = 65%; Figure 2) and lumbar disc herniation (*n* = 355; SMD: −0.51; 95% CI −0.83 to −0.20; *P* = 0.001; heterogeneity: *χ*^2^ = 3.45, *P* = 0.18, *I*^2^ = 42%; Figure 2), both combined (*n* = 829; SMD: −0.64; 95% CI −0.87 to −0.40; *P* < 0.00001; heterogeneity: *χ*^2^ = 20.45, *P* = 0.009, *I*^2^ = 61%; Table 2, Figure 2).

*(3) Effects of C(T)MT versus Physical Therapies on Pain*. Three RCTs examined the effect of C(T)MT versus physical therapies on pain relief [5, 69, 80]. All of these RCTs were included in the meta-analysis. The meta-analysis showed the superior effect of C(T)MT on pain relief (*n* = 214; WMD: −0.97; 95% CI −1.46 to −0.48; *P* < 0.0001; heterogeneity: *χ*^2^ = 2.96, *P* < 0.23, *I*^2^ = 32%; Table 2).

*(4) Effects of C(T)MT versus Drugs on Pain*. Among the 5 RCTs that assessed the effect of C(T)MT versus drugs on pain relief, three studies researched the effects on spine condition disorders. They focused on different locations: lumbar [67], cervical [42], and overall spine [71]. Moreover, the method of C(T)MT also differed from acupoint C(T)MT [42, 71] to general C(T)MT [67]. The aggregated results suggested that C(T)MT produced similar effects on pain when compared with drugs (*n* = 728; WMD, −0.46; 95% CI −1.05 to 0.13; *P* = 0.13; heterogeneity: *χ*^2^ = 17.02, *P* = 0.0002, *I*^2^ = 88%; Figure 2). Another 2 studies assessed the effects on musculoskeletal disorders of the extremities [83, 85]. The meta-analysis for these 2 did not show any superior effect of C(T)MT on pain (*n* = 166; WMD: −0.41; 95% CI −0.90 to 0.08; *P* = 0.10; heterogeneity: *χ*^2^ = 0.16, *P* = 0.69, *I*^2^ = 0%; Figure 2). However, when 5 studies were examined together through statistical pooling, the results showed favourable effects of C(T)MT on pain, but this was heterogeneous (*n* = 848; WMD: −0.44; 95% CI −0.85 to 0.02; *P* = 0.04; heterogeneity: *χ*^2^ = 17.27, *P* = 0.002, *I*^2^ = 77%; Figure 2).

*(5) Effects of C(T)MT Plus Traction versus Traction on Pain*. A total of 3 studies were available for statistical pooling (Figure 2 and Table 2). Two of them focused on diseases of the lumbar region [91, 93], and the last one looked at the cervical region [89]. The meta-analysis showed favourable effects of C(T)MT plus traction on pain reduction (*n* = 190; WMD: −1.08; 95% CI −1.81 to −0.35; *P* = 0.004; Table 2). However, they also showed high heterogeneity (heterogeneity: *χ*^2^ = 43.23, *P* < 0.00001, *I*^2^ = 95%; Table 2).

*(6) Effects of C(T)MT Plus Drugs versus Drugs Alone on Pain*. Six RCTs compared the effects of C(T)MT plus drugs on pain to the effects of drugs only (Figure 2 and Table 2). Among them, 2 RCTs involved lumbar disc herniation [90, 92] and the other 2 RCTs involved unspecified spinal diseases such as scoliosis [96] and ankylosing spondylitis [97]. The remaining 2 RCTs involved scapulohumeral periarthritis that we classified as extremity diseases [98, 99]. When all studies were analysed in the meta-analysis, the results were favourable but with high heterogeneity (*n* = 442; WMD: −0.99; 95% CI −1.70 to −0.28; *P* = 0.006; heterogeneity: *χ*^2^ = 53.71, *P* < 0.00001, *I*^2^ = 91%; Figure 2 and Table 2). The subgroup analysis revealed the following results. The meta-analysis for the first group of RCTs did not show any favourable effects of C(T)MT plus drugs on pain reduction (*n* = 124; WMD, −0.16; 95% CI, −0.64 to 0.33; *P* = 0.53; heterogeneity: *χ*^2^ = 0.01, *P* = 0.94, *I*^2^ = 0%; Figure 2). For the second group of RCTs, the meta-analysis showed favourable effects of C(T)MT plus drugs on pain reduction (*n* = 120; WMD: −0.83; 95% CI −1.12 to −0.55; *P* < 0.00001; heterogeneity: *χ*^2^ = 0.32, *P* = 0.57, *I*^2^ = 0%; Figure 2). The last group of RCTs appeared to show favourable effects of C(T)MT plus drugs on pain reduction in the meta-analysis (*n* = 198; WMD: −1.86; 95% CI −2.79 to −0.92; *P* = 0.0001; heterogeneity: *χ*^2^ = 6.10, *P* = 0.01, *I*^2^ = 84%; Figure 2). As shown, only the last group was found to have high heterogeneity.

*(7) Effects of C(T)MT Plus Surgery versus Surgery on Pain*. Two RCTs tested the effects of C(T)MT plus surgical intervention on pain for vertebral fractures and compared this with the effects of surgical intervention only [94, 95]. The meta-analysis did not show favourable effects of C(T)MT plus surgical intervention on pain reduction (*n* = 92; WMD: −0.47; 95% CI −1.60 to 0.66; *P* = 0.41; Figure 2). The results also showed signs of heterogeneity (heterogeneity: *χ*^2^ = 9.63, *P* = 0.02, *I*^2^ = 90%; Figure 2).

#### 3.4.2. Effects of C(T)MT on Function Status

*(1) Effects of C(T)MT versus Traction on Neck Function*. Three studies that compared C(T)MT with traction for the improvement of NDI score [50, 60, 61] reported that C(T)MT was not more effective than traction (*n* = 226; SMD −1.45, 95% CI: −2.92 to 0.02, *P* = 0.05; heterogeneity: *I*^2^ = 96%; Figure 2).

*(2) Effects of C(T)MT Plus Drug versus Drugs on Low Back Function*. Of 3 studies about improvements in low back function status, 2 used IV injection to treat lumbar spine [90, 92] and one used oral drugs to treat scoliosis [96]. Treatment with C(T)MT and drugs slightly improved ODI scores compared to drug treatment only (*n* = 184; SMD −1.79, 95% CI: −3.54 to −0.04, *P* = 0.04; heterogeneity: *I*^2^ = 96%; Figure 2).

#### 3.4.3. Effects of C(T)MT on Both of Pain and Function Status

*(1) Effects of C(T)MT versus Surgery on Shoulder Pain and Function*. Regarding shoulder pain and function degradation caused by humeral fractures [75, 76], the Constant-Murley score did not show a significant difference between a C(T)MT and a surgical intervention group (*n* = 158; WMD 3.33, 95% CI: −4.59 to −11.25, *P* = 0.41; heterogeneity: *I*^2^ = 99%; Figure 2).

#### 3.4.4. Incidence of Complications

Only 7 studies reported on the incidence of complications. In 5 studies, they compared C(T)MT with surgical interventions and reported complications, such as impaired wound healing, nerve or tendon injury, infection, and traumatic arthritis in C(T)MT and surgery group [76, 78, 79, 86, 87]. The meta-analysis showed favourable effects of C(T)MT on the incidence of complications (*n* = 384; RR 0.45, 95% CI: 0.26 to 0.76, *P* = 0.003; heterogeneity: *I*^2^ = 0%; Figure 2). Although one study that compared C(T)MT with surgery for surgical neck of humerus fractures reported a statistically significant difference between C(T)MT and surgery groups (*P* < 0.05), no specific data were assessable [77]. One study that compared C(T)MT with conservative treatment for acromioclavicular joint dislocation reported complications such as joint dysfunction and nerve and vascular injury. On the incidence of complications, the C(T)MT group had a lower complication rate than the control group and this variance was significantly different (*P* < 0.05) [74]. The other 59 trials did not mention complications.

## 4. Discussion

The purpose of our systematic review was to evaluate the current evidence of the effectiveness of C(T)MT for patients with musculoskeletal disease. As a main finding, we found meaningful evidence of the effectiveness of C(T)MT on pain reduction through our meta-analyses. Although our analyses included only 1 sham-controlled RCT comparing C(T)MT to sham C(T)MT [40], this study showed that C(T)MT has an immediate effect on pain relief. Other studies included in our review also showed that the effectiveness of C(T)MT on lessening pain was better than traction, drugs, and physical therapies. In studies where the effects of both C(T)MT and other interventions (e.g., tractions, drugs, and surgery) were compared with other same interventions only, the analysis demonstrated that the combination of both was better at improving pain except when combined with surgical interventions.

The meta-analysis also looked at 6 RCTs on improvement of functional status. In studies where drugs were given to both groups and C(T)MT to the experimental group, the improvement of low back function was shown to be favourable [90, 92, 96]. When the effects of C(T)MT on neck function were compared to the effects of traction, the results were not statistically different, and the treatments had similar effects on improvement of low back function [50, 60, 61].

To explore the impact of C(T)MT on musculoskeletal diseases through pain reduction and functional improvement, the meta-analysis included only studies with adequate randomization. By doing this, a large number of quasi-RCTs (*n* = 321) were excluded to prevent selection bias. More importantly, this process suggested that this particular meta-analysis was capable of demonstrating proper evidence of the effectiveness of C(T)MT on musculoskeletal diseases. Furthermore, since the result of statistical pooling showed that C(T)MT was meaningfully effective for treating pain, except when compared to surgery, this meant that C(T)MT had similar/or equal effects on pain reduction to traction, drugs, or physical therapy to treat musculoskeletal diseases. Moreover, prescribing C(T)MT with other treatments could potentially result in better treatments than sole treatments. The results of statistical pooling on functional improvement also showed meaningful results, but evidence for functional improvement was not as strong as that for pain reduction. However, the meta-analysis was based mainly on small-sized experiments and diverse interventions were used in clinical trials. Therefore, the results should be carefully interpreted.

Furthermore, our analysis assumed that C(T)MT did not cause serious complications compared to other interventions [76, 78, 79, 86, 87]. Several mild to severe adverse events have been previously reported [106], but they may be regarded as rare.

Previously, there were clinical guidelines or systematic reviews of manual therapies for lumbar or cervical disease. The clinical guidelines in two countries, the United States in 2007 [107] and the UK in 2009 [108], reported moderate-quality evidence to support the potency of massage and spinal manipulation in the treatment of LBP. Additionally, a systematic review including 13 RCTs reported potential benefits of massage to reduce pain from subacute and chronic nonspecific LBP [18]. Another systematic review based on 15 RCTs reported that MT had a better immediate effect on pain relief than inactive therapies [20]. An additional systematic review of 7 RCTs, published in 2013, showed that MT was more effective than inactive therapies for neck and shoulder pain, but there was no evidence of an improvement in functional status from MT [15]. However, all of these studies and guidelines analysed the effect of MT by looking not only at C(T)MT, but also at common Western massage, traditional Thai massage, classical strain/counterstrain technique, myofascial band therapy, and so on. Therefore, it was difficult to pinpoint the specific effect of C(T)MT. Very rarely, there were studies that focused on the C(T)MT only. Wei et al. [109] reported that C(T)MT resulted in better pain relief than computer traction on cervical radiculopathy. In addition, a systematic review of 13 RCTs reported that the combination of C(T)MT and Chinese medicine or acupuncture was effective for pain relief and functional improvement of LBP [16].

We analysed all RCTs that investigated the effects of C(T)MT on any musculoskeletal disorders published worldwide until December 31, 2016. The results helped to set priorities and directions for future research on C(T)MT by analysing all studies, regardless of the kind of disease. More specifically, once we collected all studies on C(T)MT, we took steps to divide collected studies into subgroups to provide a clearer picture on the present state of studies on C(T)MT. This was an unprecedented type of study. Additionally, we confined our research to traditional Chinese and Korean manual techniques by limiting interventions to Chuna and Tuina to clarify the effects of C(T)MT. By focusing on qualified RCTs, we managed to categorize a large volume of quantitative and qualitative data on the in depth assessment of C(T)MT with regard to pain and function in musculoskeletal diseases. We also sought to suggest the wide range of applicability of C(T)MT. We classified all studies with various control groups into three designs such as C(T)MT versus Sham C(T)MT, C(T)MT versus OIs, and C(T)MT plus OIs versus OIs to suggest alternative or cooperative treatments for C(T)MT.

Our meta-analysis had some limitations. Even though we searched through numerous databases and collected published studies from the US, the EU, China, Japan, and South Korea, all studies except seven were written in Chinese and published in Chinese journals that were not registered in Medline. Seven remaining studies were published in journals that were indexed in Medline. One of them was written in English and conducted in Portugal [40], and others were written in Chinese and performed in China [5, 42, 63, 71, 72, 104]. Since it has been reported that studies written in non-English languages and published in journals that are not listed in Medline have the potential to inflate the effect estimates [110], our analysis might have been influenced by language bias.

Moreover, out of 66 studies analysed in this review, there was only one study that included sham C(T)MT [40]. Consequently, this situation limited our ability to exactly evaluate the size of effects of C(T)MT. However, this limitation is likely caused by the nature of C(T)MT.

Most studies included in this study had methodological weaknesses. Of 66 RCTs with adequate randomization, only 18 of these studies (27.3%) managed to have appropriate allocation concealment. This is concerning for two reasons. The overestimation of treatment effects is known to be caused by inadequate allocation concealment or random sequence generation [38, 39] and the most important source of bias in RCTs is unconcealed allocation [111]. Another limitation was caused by serious flaws in the blinding methods used in most RCTs. In C(T)MT, it is impossible to blind the therapists and hard to blind the subjects. To overcome this problem, blinded assessors and concealed allocation should have been implemented. However, most RCTs failed to carry out these compensating methods and only 2 of 66 RCTs (3%) were assessor blinded. Therefore, the outcome data from these studies might have been overestimated.

Fortunately, studies in our review had comparatively good average sample sizes per arm: 46.7 in the treatment groups and 45.2 in the control groups. Moore et al. [112] reported that it was necessary to have at least 40 per arm to earn meaningful results in a clinical trial on pain based on the simulation they performed. Even though our review included studies with small sample sizes, the average sample size of all studies turned out to be big enough to ensure validity.

Additionally, the clinical heterogeneities of some of our meta-analyses might limit the translation of our results [113]. We believe that the existence of heterogeneity is due to diverse methods of C(T)MT. Additionally, the studies we considered tested various drugs and surgery methods and differed in duration of treatments offered and diseases studied.

Since the review included all musculoskeletal conditions/diseases, we were mindful of the possibility that the focus of our review might seem unclear. Therefore, we made extra efforts to increase the statistical/clinical homogeneity. To do so, we tried to find studies that matched perfectly with one another in PICO: population, intervention, comparison, and outcomes. However, the number of studies satisfying this requirement was too small. Therefore, discussing comparative effects between particular treatments in control groups requires a cautious approach. Although it presents a very difficult methodological problem, sham research should be continued and a comparative effectiveness study also is recommended.

This review demonstrated the possibilities of using C(T)MT through clinical applicability, but we did not consider analysing the standardization of C(T)MT. The lack of standardization may be due to the fact that the effectiveness of C(T)MT can be influenced by many variables, including C(T)MT techniques, application of time duration for each treatment and the number of treatments, their lengths and repeats. In this review, many of these variables were present in most studies, and they were widely heterogeneous on clinical factors. To move forward, future studies should not only carry out investigations into the effectiveness and safety of C(T)MT but also investigate the effectiveness of interventions based on standardized guidelines.

## 5. Conclusions

Our systematic review of 66 RCTs demonstrated that C(T)MT might have favourable effects on pain and functional improvements caused by musculoskeletal diseases, but the evidence for functional improvement was not as strong as for pain relief. Moreover, this study indicated that C(T)MT is a safe intervention. However, given the low quality of the included studies and the diverse methods of intervention techniques, the available evidence is insufficient to determine the effects of C(T)MT. In conclusion, to prove the effects of C(T)MT on the pain and dysfunction associated with musculoskeletal disease, high-quality RCTs such as sham-controlled studies with standardized interventions are needed.

## Figures and Tables

**Table 1:** Categories of musculoskeletal conditions and number of randomized controlled trials (*n*).

Musculoskeletal conditions	Number of studies				
	C(T)MT versus sham	C(T)MT versus OIs	C(T)MT + OIs vs. OIs	Number	Total
*Spine*					
Cervical	26.6%				
Cervical spondylotic radiculopathy		11	1	12	
Cervical spondylosis		7		7	
Lower cervical vertebral degenerative instability		2		2	
Atlantoaxial joint disorder		1		1	
Curvature abnormality			1	1	
Cervical shoulder pain		1		1	
		**22**	**2**		**24**

Thoracolumbar	24.3%				
Lumbar disc herniation		5	4	9	
Lumbar muscle strain		3		3	
Thoracolumbar fracture			2	2	
		**8**	**6**		**14**

Others					
Scoliosis		1	1	2	
Sacrococcygeal pain		1		1	
Ankylosing spondylitis			1	1	
		**2**	**2**		**4**

*Upper Extremity*					
Shoulder	27.3%				
Periarthritis of shoulder		2	2	4	
Acromioclavicular dislocation		1		1	
		**3**	**2**		**5**

Arm and hand					
Humeral fracture		3		3	
Radius fracture		2		2	
Lateral epicondylitis of humerus		1	1	2	
Brachial plexus block		1		1	
		**7**	**1**		**8**

*Lower Extremity*					
Knee					
Knee osteoarthritis		4	1	5	
Post knee surgery pain or dysfunction			2	2	
Kaschin-Beck disease			1	1	
		**4**	**4**		**8**

Leg and foot					
Calcaneal fracture		1		1	
Ankle fracture		1		1	
		**2**			**2**

Total	**1**	**48**	**17**		**66**

C(T)MT: Chuna (or Tuina) manual therapy; OIs: other interventions.

**Table 2:** Effect estimates of C(T)MT for pain and function on musculoskeletal conditions.

Outcomes	Number of studies^ref^	Number of patients	Effect estimate [95% CI]	*P* value	*I* ^2^ (%)
*Pain intensity (VAS or NRS)*					
C(T)MT versus sham	1 [40]	69	SMD −3.09 [−3.59, −2.59]	*P* = 0.00001	NA
C(T)MT versus traction	9 [44, 46, 49–51, 60, 63–65]	829	SMD −0.64 [−0.87, −0.40]	*P* < 0.00001	61
C(T)MT versus physical therapy	3 [5, 69, 80]	214	WMD −0.97 [−1.46, −0.48]	*P* < 0.0001	32
C(T)MT versus drug	5 [42, 43, 67, 71, 85]	848	WMD −0.44 [−0.85, −0.02]	*P* = 0.04	77
C(T)MT + traction versus traction	3 [89, 91, 93]	190	WMD −1.08 [−1.81, −0.35]	*P* = 0.004	95
C(T)MT + drug versus drug	6 [90, 92, 96–99]	442	WMD −0.99 [−1.70, −0.28]	*P* = 0.006	91
C(T)MT + surgery versus surgery	2 [94, 95]	92	WMD −0.47 [−1.60, 0.66]	*P* = 0.41	90

*Neck function (NDI)*					
C(T)MT versus traction	3 [50, 60, 61]	226	SMD −1.45 [−2.92, 0.02]	*P* = 0.05	96

*Low back function (ODI)*					
C(T)MT + drug versus drug	3 [90, 92, 96]	184	SMD −1.79 [−3.54, −0.04]	*P* = 0.04	96

*Shoulder pain and function (CMS)*					
C(T)MT versus surgery	2 [75, 76]	158	WMD 3.33 [−4.59,11.25]	*P* = 0.41	99

*Complication*					
C(T)MT versus surgery	5 [76, 78, 79, 86, 87]	384	RR 0.45 [0.26, 0.76]	*P* = 0.003	0

ref: reference; CMS: Constant-Murley score; C(T)MT: Chuna (or Tuina) manual therapy; NA: not applicable; NDI: neck disability index; ODI: Oswestry disability index; RR: relative risk; SMD: standard mean difference; VAS: visual analogue scale; WMD: weight mean difference.

**Table 3:** Summary of randomized controlled trials of C(T)MT for pain and function for musculoskeletal diseases.

Author (Year) Country	Types of disease	Design	Sample size (A/B/C)	Duration weeks	Follow-up weeks	Intervention		Outcome measures	Main results
						Treatment	Control		
*C(T)MT vs. sham C(T)MT (1)*									
Sousa et al. [40] (2015) Portugal	Working-related musculoskeletal disorder of professional orchestra musicians	Patient blind, parallel 2 arms	69 (39/30)	Immediate effect	nr	C(T)MT (real acupoints)	Sham C(T)MT (nonspecific skin points)	(1) VNRS (pain)	(1) positive^b^

*C(T)MT vs. OIs (48)*									
Zhu et al. [41] (2007) China	Cervical spondylotic radiculopathy	Parallel 2 arms	116 (59/57)	4	nr	C(T)MT (8 sessions)	TR (30 min/8 sessions)	(1) VAS	(1) Positive^a^

Chen et al. [42] (2009) China	Cervical spondylotic radiculopathy	Parallel 2 arms	400 (200/200)	34 d	nr	C(T)MT (30 sessions)	TCM (tid/30 sessions)	(1) VAS (2) ADL	(1) Positive^b^ (2) Positive^b^

Wang et al. [43] (2009) China	Cervical spondylotic radiculopathy	Parallel 2 arms	110 (54/56)	2	4	C(T)MT (7 sessions)	TR (30 min/14 sessions)	(1) VAS (2) ROM	(1) Positive^a^ (2) NA

Huang [44] (2010) China	Cervical spondylotic radiculopathy	Parallel 2 arms	60 (30/30)	4	6 m	C(T)MT (20 min/28 sessions)	TR (30 min/28 sessions)	(1) VAS (2) TSS ① Neck pain and discomfort ② Upper limb pain and numbness ③ ADL ④ Distal sensory strength ⑤ Upper limb tension reflex weakness ⑥ Spurling's test	(1) Positive^a^ (2) TSS ① Positive^a^ ② Positive^a^ ③ Positive^a^ ④ Positive^a^ ⑤ Positive^a^ ⑥ Positive^a^

Liao et al. [45] (2011) China	Cervical spondylotic radiculopathy	Parallel 2 arms	111 (56/55)	4	nr	C(T)MT (8 sessions)	CPD (tid)	(1) MPQ	(1) Positive^a^

Jiang et al. [46] (2012) China	Cervical spondylotic radiculopathy	Parallel 2 arms	79 (38/41)	2	nr	C(T)MT (7 sessions)	TR (20 min/14 sessions)	(1) VAS	(1) Positive^b^

Qin et al. [47] (2012) China	Cervical spondylotic radiculopathy	Parallel 2 arms	60 (30/30)	2	nr	C(T)MT (8 sessions)	TR (20 min/8 sessions)	(1) TSS ① Neck pain and discomfort ② Upper limb pain and numbness ③ ADL ④ Distal sensory strength ⑤ Upper limb tension reflex weakness ⑥ Spurling's test	(1) TSS ① Positive^a^ ② Positive^a^ ③ NS ④ Positive^a^ ⑤ NS ⑥ Positive^a^

Xu [48] (2013) China	Cervical spondylotic radiculopathy	Parallel 2 arms	36 (18/18)	4	nr	C(T)MT (12 sessions)	TR (20–30 min/12 sessions)	(1) VAS (2) TSS	(1) Positive^a^ (2) Positive^a^

Xue [49] (2015) China	Cervical spondylotic radiculopathy	Parallel 2 arms	130 (65/65)	2	nr	C(T)MT (nr)	TR (15 min/14 sessions)	(1) VAS (2) NDI	(1) Positive^a^ (2) Positive^a^

Yang [50] (2015) China	Cervical spondylotic radiculopathy	Parallel 2 arms	72 (36/36)	2	nr	C(T)MT (30 min/6 sessions)	TR (20 min/6 sessions)	(1) VAS (2) NDI (3) SF-36	(1) Positive^a^ (2) Positive^a^ (3) Positive^a^

Liu [51] (2015) China	Cervical spondylotic radiculopathy	Parallel 3 arms	114 (38/38/38)	NA	1 m	C(T)MT (30 min/4 sessions)	B: TR (4 sessions) C: MT (4 sessions)	(1) VAS (2) ROM	(1) Positive^b^ (2) NS (1 w: Positive^a^, 1 m: Positive^b^)

Zhu et al. [52] (2009) China	Cervical spondylosis	Parallel 2 arms	210 (106/104)	2	1 m	C(T)MT (7 sessions)	TR (30 min/7 sessions)	(1) VAS	(1) Positive^c^

Lin et al. [53] (2012) China	Cervical spondylosis	Parallel 2 arms	70 (35/35)	4	nr	C(T)MT (12 sessions)	CPD (tid)	(1) NRS (2) ROM (3) MMS	(1) Positive^a^ (2) Positive^a^ (3) Positive^a^

Yan et al. [54] (2014) China	Cervical spondylosis	Parallel 2 arms	70 (36/34)	2	nr	C(T)MT (20 min/6 sessions)	TCM external preparation (qd/6 sessions)	(1) VAS (2) CASCS	(1) Positive^a^ (2) Positive^a^

Li and Zhou [55] (2016) China	Cervical spondylosis (sympathetic nerve type)	Parallel 2 arms	80 (40/40)	2	nr	C(T)MT (10 sessions)	OD	(1) CASCS (2) JOA	(1) Positive^b^ (2) NS

Jin [56] (2008) China	Cervical spondylosis (vertebral artery type)	Parallel 2 arms	55 (27/28)	2	3 m	C(T)MT (7 sessions)	TR (30 min/14 sessions)	(1) TSS (2) IC	(1) Positive^a^ (2) NS

Gao et al. [57] (2011) China	Cervical spondylosis (vertebral artery type)	Parallel 2 arms	177 (87/90)	14 d	nr	C(T)MT (7 sessions)	TR (30 min/14 sessions)	(1) ROM	(1) Positive^c^

Zeng [58] (2015) China	Cervical spondylosis (vertebral artery type)	Parallel 3 arms	45 (15/15/15)	4	nr	C(T)MT (30 min/8 sessions)	B: MCT (8 sessions) C: C(T)MT plus MCT	(1) VAS (2) TSS	(1) NA (2) NA

Sun [59] (2007) China	Atlantoaxial joint disorder	Parallel 2 arms	93 (55/38)	2	1 m	C(T)MT	TR (30 min/7 sessions)	(1) TSS	(1) Positive^b^

Wang [60] (2012) China	Lower cervical vertebral degenerative instability	Parallel 2 arms	60 (30/30)	2	1 m	C(T)MT (10 sessions)	TR (20 min/10 sessions)	(1) VAS (2) NDI	(1) Positive^a^ (2) Positive^a^

Yang et al. [61] (2014) China	Lower cervical vertebral degenerative instability	Parallel 2 arms	97 (49/48)	2	nr	C(T)MT (6 sessions)	TR (20 min/10 sessions)	(1) NDI	(1) Positive^b^

Chen et al. [62] (2011) China	Cervical shoulder pain	Parallel 2 arms	68 (35/33)	6 d	nr	C(T)MT (30 min/6 sessions)	OD (bid)	(1) VAS	(1) Positive^b^

Chen et al. [63] (2006) China	Lumbar disc herniation	Parallel 2 arms	233 (122/111)	4	nr	C(T)MT (15 min/8 sessions)	TR (20 min/15 sessions)	(1) VAS (2) ROM (3) SLR (4) M-JOA	(1) Positive^b^ (2) Positive^a^ (3) NS (4) Positive^b^

Wang [64] (2010) China	Lumbar disc herniation	Parallel 2 arms	62 (32/30)	3	nr	C(T)MT (every second day)	TR (every day)	(1) VAS (2) JOA (3) SLRT	(1) Positive^b^ (2) Positive^b^ (3) Positive^b^

Zhou et al. [65] (2012) China	Lumbar disc herniation	Parallel 2 arms	65 (32/33)	12 d	nr	C(T)MT (20 min/6 sessions)	TR (20 min/10 sessions)	(1) VAS (2) ODI	(1) Positive^b^ (2) Positive^a^

Luo et al. [66] (2013) China	Lumbar disc herniation	Parallel 2 arms	60 (30/30)	1 m		C(T)MT (8 sessions)	TR (30 min/14 sessions)	(1) VAS (2) JOA (3) SF-36 ① PF ② RP ③ BP ④ SF ⑤ MH ⑥ RE ⑦ VT ⑧ GH	(1) Positive^a^ (2) Positive^b^ (3) Positive^b^ ① Positive^a^ ② Positive^b^ ③ Positive^b^ ④ Positive^a^ ⑤ Positive^b^ ⑥ Positive^b^ ⑦ NS ⑧ Positive^a^

Deng et al. [67] (2012) China	Lumbar disc herniation	Parallel 2 arms	290 (145/145)	2	A: 3 w B: 1 y	C(T)MT (every second day)	OD (qd)	(1) VAS	(1) A: NS B: Positive^a^

Zhang et al. [68] (2005) China	Lumbar muscle strain	Parallel 2 arms	105 (51/54)	5–7 d	nr	C(T)MT (30 min/5 sessions)	OD	(1) ALBP clinical score	(1) Positive^b^

Xue [69] (2016) China	Lumbar muscle strain	Parallel 2 arms	63 (32/31)	3-4	3 m	C(T)MT (30 min/10 sessions)	TR (20 min/10 sessions)	(1) VAS	(1) Positive^a^

Zhang [70] (2010) China	Lumbar muscle strain	Parallel 2 arms	62 (31/31)	8	nr	C(T)MT (30 min/24 sessions)	CPD (bid)	(1) Symptom score (2) Sign score	(1) NA (2) NA

Tian et al. [71] (2015) China	Degenerative scoliosis	Parallel 2 arms	38 (20/18)	10–15	nr	C(T)MT (30 min/36 sessions)	OD	(1) VAS (2) ODI	(1) Positive^a^ (2) Positive^a^

Wang et al. [72] (2016) China	Sacrococcygeal pain	Parallel 2 arms	184 (91/93)	2	3 m	C(T)MT (6 sessions)	EM (external medicine) (bid)	(1) VAS (2) Rating scale of sacrococcygeal pain	(1) Positive^c^ (2) Positive^c^

Wang et al. [73] (2013) China	Periarthritis of shoulder	Parallel 2 arms	120 (60/60)	20 d	nr	C(T)MT (20 sessions)	TCM external preparation	(1) SF-36 ① PF ② RP ③ BP ④ SF ⑤ MH ⑥ RE ⑦ VT ⑧ GH	(1) Positive^b^ ① Positive^b^ ② Positive^a^ ③ Positive^b^ ④ NS ⑤ Positive^b^ ⑥ Positive^a^ ⑦ Positive^a^ ⑧ Positive^a^

Chen et al. [5] (2013) China	Periarthritis of shoulder	Parallel 3 arms	120 (40/40/40)	4	nr	C(T)MT (24 sessions)	B: EA (24 sessions) C: PT (TENS) (24 sessions)	(1) VAS (2) ROM	(1) Positive^a^ (2) Positive^a^

Xu [74] (2014) China	Acromioclavicular dislocation	Parallel 2 arms	120 (60/60)	NA	nr	C(T)MT	PT (upper limb abduction splint)	(1) IC	(1) Positive^a^

Xu [75] (2016) China	Humeral fracture	Parallel 2 arms	94 (47/47)	7 d	7 d	C(T)MT	Surgery	(1) CMS ① Pain ② Function ③ ROM ④ Muscle strength	(1) CM ① Positive^b^ ② Positive^b^ ③ Positive^b^ ④ Positive^b^

Pan [76] (2016) China	Humeral fracture	Parallel 2 arms	64 (32/32)	6	12 m	C(T)MT	Surgery	(1) CMS (2) IC	(1) NS (2) Positive^a^

Yang [77] (2004) China	Humeral fracture	Parallel 2 arms	68 (34/34)	6	nr	C(T)MT	Surgery	(1) IC (2) ROM	(1) 2 w: NS 4 w: Positive^a^ 6 w: Positive^a^ (2) 2 w: NA 4 w: Positive^a^ 6 w: Positive^a^

Pan [78] (2015) China	Radius fracture	Parallel 2 arms	60 (30/30)	NA	nr	C(T)MT	Surgery	(1) IC	(1) Positive^a^

Li [79] (2016) China	Radius fracture	Parallel 2 arms	80 (48/32)	NA	NA	C(T)MT	Surgery	(1) Recovery rate of joint function (2) Time ① Swelling subside ② Fracture healing ③ Pain subside (3) IC	(1) Positive^b^ (2) Time ① Positive^a^ ② Positive^a^ ③ Positive^a^ (3) Positive^a^

Ding et al. [80] (2010) China	Lateral epicondylitis of humerus	Parallel 2 arms	76 (38/38)	2	nr	C(T)MT	PT (IFT) (14 sessions)	(1) VAS (2) Mayo Score	(1) Positive^b^ (2) Positive^a^

Gao and Yan [81] (2014) China	Brachial plexus block	Parallel 2 arms	200 (120/80)	3	3 m	C(T)MT (3 sessions)	BT (3–9 sessions)	(1) VAS (2) Melle Score	(1) Positive^a^ (2) Positive^a^

Tian [82] (2010) China	Knee osteoarthritis	Parallel 2 arms	60 (30/30)	31 d	nr	C(T)MT (20 min/30 sessions)	CPD (bid)	(1) MPQ-SF (2) JOA	(1) Positive^a^ (2) Positive^a^

Chen [83] (2015) China	Knee osteoarthritis	Parallel 2 arms	60 (30/30)	1 m	nr	C(T)MT (12 sessions)	OD (qd)	(1) WOMAC (2) VAS	(1) Positive^a^ (2) NS

Jin [84] (2015) China	Knee osteoarthritis	Parallel 2 arms	120 (60/60)	4	nr	C(T)MT (12 sessions)	OD (qd)	(1) WOMAC ① Pain ② Stiffness ③ Physical Function	(1) WOMAC ① NS ② Positive^a^ ③ Positive^a^

Li et al. [85] (2016) China	Knee osteoarthritis	Parallel 2 arms	60 (30/30)	4	nr	C(T)MT (20 sessions)	OD (bid)	(1) VAS (2) JOA	(1) Positive^a^ (2) Positive^a^

Ren [86] (2014) China	Ankle fracture, trimalleolar fracture	Parallel 2 arms	110 (55/55)	NA	nr	C(T)MT	Surgery	(1) IC	(1) Positive^a^

Zhao et al. [87] (2016) China	Calcaneal Fracture	Parallel 2 arms	66 (34/32)	NA	9–15 m	C(T)MT	Surgery	(1) IC (2) Fracture healing time (3) AOFAS scale ① Pain ② ADL ③ X-ray	(1) Positive^a^ (2) NS (3) NS ① NS ② NS ③ NS

*C(T)MT + OIs versus OIs (17)*									
Chen and Tang [88] (2013) China	Cervical spondylotic radiculopathy	Parallel 2 arms	60 (30/30)	3	nr	C(T)MT plus TR (15 sessions)	TR (15 sessions)	(1) VAS (2) PRI (3) PPI	(1) Positive^a^ (2) Positive^a^ (3) Positive^a^

Zhang and Hai [89] (2016) China	Curvature abnormality	Parallel 2 arms	70 (36/34)	2	nr	C(T)MT plus TR (14 sessions)	TR (14 sessions)	(1) VAS	(1) Positive^a^

Dong and Wang [90] (2014) China	Lumbar disc herniation	Parallel 2 arms	80 (40/40)	30 d	nr	C(T)MT plus OD (tid), IV (qd)	OD (tid), IV (qd)	(1) ODI (2) JOA (3) VAS	(1) Positive^a^ (2) Positive^a^ (3) NS

Song et al. [91] (2015) China	Lumbar disc herniation	Parallel 2 arms	60 (30/30)	10 d	nr	C(T)MT plus TR	TR	(1) VAS (2) JOA	(1) Positive^a^ (2) Positive^a^

Yin et al. [92] (2015) China	Lumbar disc herniation	Parallel 2 arms	50 (25/25)	2	6 m	C(T)MT plus IV (qd)	IV (qd)	(1) VAS (2) ODI	(1) Positive^a^ (2) Positive^a^

Wu et al. [93] (2016) China	Lumbar disc herniation	Parallel 2 arms	60 (30/30)	10 d	nr	C(T)MT plus TR (10 sessions)	TR (10 sessions)	(1) VAS	(1) Positive^c^

Zhang et al. [94] (2016) China	Thoracolumbar fracture	Parallel 2 arms	40 (20/20)	1 d	1 d, 3 d	C(T)MT plus Surgery	Surgery (PPF)	(1) VAS	(1) Positive^a^

Yu et al. [95] (2016) China	Thoracolumbar fracture	Parallel 2 arms	52 (26/26)	1 d	3 d, 2 w, 6 m	C(T)MT plus Surgery	Surgery (PKP)	(1) VAS	(1) NS

Sun et al. [96] (2016) China	Degenerative Scoliosis	Parallel 2 arms	60 (30/30)	3	nr	C(T)MT plus OD	OD (tid)	(1) VAS (2) ODI	(1) Positive^c^ (2) Positive^b^

Jia and Sha [97] (2015) China	Ankylosing spondylitis	Parallel 2 arms	60 (30/30)	4	nr	C(T)MT plus OD	OD (tid)	(1) VAS (2) Metrology index of bath ankylosing spondylitis	(1) Positive^a^ (2) Positive^a^

Zhang [98] (2015) China	Periarthritis of shoulder	Parallel 2 arms	78 (39/39)	4	nr	C(T)MT (28 sessions) plus PP (4 sessions)	PP (4 sessions)	(1) VAS (2) Symptom score ① Shoulder pain ② ROM limitation ③ Shoulder coldness ④ Shoulder muscular atrophy ⑤ Numbness and weakness	(1) Positive^a^ (2) Symptom Score ① Positive^a^ ② Positive^a^ ③ Positive^a^ ④ Positive^a^ ⑤ Positive^a^

Shen et al. [99] (2015) China	Periarthritis of shoulder	Parallel 2 arms	120 (60/60)	1 m	nr	C(T)MT plus PP	PP (3 sessions)	(1) VAS	(1) Positive^a^

Wu [100] (2011) China	Lateral epicondylitis of humerus	Parallel 2 arms	22 (11/11)	9 d	nr	C(T)MT plus PT (UT + IFT) (5 sessions)	PT (UT + IFT) (5 sessions)	(1) VAS	(1) Positive^a^

Xiao [101] (2016) China	Knee osteoarthritis	Parallel 2 arms	70 (35/35)	4	nr	C(T)MT plus RT	RT	(1) HSS Score ① Pain ② Knee function ③ ROM ④ Knee flexion Deformity ⑤ Stability ⑥ Muscle strength (2) SF-36 ① PF ② RP ③ BP ④ SF ⑤ MH ⑥ RE ⑦ VT ⑧ GH	(1) HSS ① Positive^a^ ② Positive^a^ ③ Positive^a^ ④ Positive^a^ ⑤ Positive^a^ ⑥ Positive^a^ (2) SF-36 ① Positive^a^ ② NS ③ Positive^a^ ④ NS ⑤ Positive^a^ ⑥ NS ⑦ NS ⑧ NS

Zhang and Deng [102] (2012) China	After knee surgery pain or function	Parallel 2 arms	80 (40/40)	Immediate effect	nr	C(T)MT plus RT (20 min/1 session)	RT	(1) Pain score	(1) Positive^b^

Wang et al. [104] (2012) China	After knee surgery pain or function	Parallel 2 arms	66 (33/33)	4	nr	C(T)MT + CPM (20 sessions)	CPM	(1) WOMAC ① Pain ② Stiffness ③ Physical Function	(1) WOMAC ① NS ② NS ③ Positive^a^

Wang et al. [103] (2016) China	Kaschin-Beck disease	Parallel 2 arms	120 (60/60)	6 m	nr	C(T)MT plus OD	OD	(1) VAS (2) ROM	(1) Positive^b^ (2) Positive^a^

^a^ *P* < 0.05; ^b^*P* < 0.01; ^c^*P* < 0.001; ADL: activities of daily living; ALBP: acute low back pain; AOFAS: American Orthopaedic Foot & Ankle Society; BP: bodily pain; BT: block therapy; CASCS: clinical assessment scale for cervical spondylosis; CMS: Constant-Murley score; C(T)MT: Chuna (or Tuina) manual therapy; CPD: Chinese patent drug; CPM: continuous Passive Training; IC: incidence of complication; IFT: intermediate frequency therapy; GH: general health; HSS: hospital for special surgery; IV: intravenous injection; JOA: joint operation agreement; MCT: microcurrent therapy; MH: mental health; MMS: maximum muscular strength; MPQ: McGill Pain Questionnaire; MPQ-SF: McGill Pain Questionnaire-Short Form; MT: manual therapy; NA: not assessable; NDI: neck disability index; NS: neutral (no significant difference between groups); OD: oral drugs; ODI: Oswestry disability index; OIs: other interventions; PP: pharmacopuncture; PF: physical functioning; PKP: percutaneous kyphoplasty; PPF: percutaneous pedicle fixation; PPI: present pain intensity; PRI: pain rating index; PT: physical therapy; RE: role-emotional; ROM: range of motion; RP: role-physical; RT: rehabilitation treatment; SF: social functioning; SF-36: short form 36 health survey; SLRT: straight leg raising test; TCM: traditional Chinese medicine; TR: traction; TSS: total score of symptoms and signs; UT: ultrasonic treatment; VAS: visual analogue scale; VNRS: visual numeric rating scale; VT: vitality; WOMAC: Western Ontario and McMaster Universities.
